# Potential Mechanism and Perspectives of Mesenchymal Stem Cell Therapy for Ischemic Stroke: A Review

**DOI:** 10.1055/s-0044-1790231

**Published:** 2024-09-02

**Authors:** Pengcheng Zhu, Hongtu Tan, Haobo Gao, Jiabin Wang, Yangyang Liu, Dongyi Yang, Tao Wu

**Affiliations:** 1 Department of Intervention, Encephalopathy Center, The First Affiliated Hospital of Henan University of Chinese Medicine, Zhengzhou, Henan Province, People's Republic of China

**Keywords:** mesenchymal stem cell, ischemic stroke, potential mechanism, self-renewal ability, chemokines

## Abstract

Mesenchymal stem cells (MSCs), as a stem cell type with multiple differentiation potentials and immune regulatory abilities, have shown broad prospects in the treatment of ischemic stroke in recent years. The main characteristics of MSCs include their self-renewal ability, differentiation potential for different types of cells, and the ability to secrete various bioactive factors such as cytokines, chemokines, and growth factors, which play a key role in tissue repair and regeneration. In the treatment of ischemic stroke, MSCs exert therapeutic effects through various mechanisms, including promoting vascular regeneration of damaged brain tissue, reducing inflammatory responses, and protecting neurons from damage caused by apoptosis. Research have shown that MSCs can promote the repair of ischemic areas by releasing neurotrophic factors and angiogenic factors, while inhibiting immune responses triggered by ischemia, thereby improving neurological function. With the in-depth study of its biological mechanism, MSCs have gradually shown good safety and effectiveness in clinical applications. Therefore, fully exploring and utilizing the potential of MSCs in the treatment of ischemic stroke may provide new ideas and solutions for future neural repair and regenerative medicine.

## Introduction

Ischemic stroke is defined as the death of brain, spinal cord, or retinal cells due to ischemia by the American Heart Association (AHA) and the American Stroke Association (ASA), which accounts for approximately 80% of the stroke population and is the main cause of disability and death, seriously affecting the quality of life in the future and bringing serious economic pressure and mental burden to patients and their families. 1 2 Stroke has become the second leading cause of death 3 and currently the leading cause of death and disability among adults in China. 4 About 70 to 80% of patients with stroke have varying degrees of disability, including paralysis, swallowing difficulties, urinary and fecal incontinence, depression, anxiety, epilepsy, dementia, cognitive dysfunction, etc. 5 Ischemic stroke is the most common type of stroke, accounting for 69.6 to 70.8% of all strokes in China. 6 During the past 15 years from 2005 to 2019, the incidence rate of ischemic stroke in China showed an overall upward trend. The incidence rate increased from 117/100,000 to 145/100,000, and the prevalence rate increased from 1,044/100,000 to 1,256/100,000. 7

Brain tissue is most sensitive to ischemia and hypoxia damage. When blood clots form, cerebral blood vessels are blocked, causing ischemia and hypoxia in brain tissue, which in turn leads to cerebral infarction and neurological dysfunction. Early restoration of blood flow perfusion is the key to the treatment of cerebral infarction. 8 9 10 At present, the main treatments for ischemic stroke include ultra early thrombolysis, anticoagulation and antiplatelet therapy, lipid-lowering and plaque stabilization, and neuroprotection. 11 12 13 Effective measures for vascular recanalization include thrombolysis and mechanical thrombectomy within the ischemic time window. However, due to limitations such as narrow treatment time windows and severe complications, only approximately 5% of patients can benefit. 14 Therefore, finding safer and more effective intervention methods has become a current research hotspot, which is of great significance for improving the neurological deficits.

Stem cells are primitive, undifferentiated cells that can develop into various specialized cells through mitosis and differentiation, with the potential to regenerate multiple tissues and organs. The emergence of stem cells and the development of stem cell transplantation technology have provided a new approach for the treatment of ischemic stroke. Various types of stem cells have been studied in animal models or clinical research, among which embryonic stem cell therapy faces ethical issues; neural progenitor cells are difficult to obtain and have a low proliferation rate; engineering cells such as induced pluripotent stem cells, human neural stem cells (NT2N/hNT), CTX0E3, SB623, etc. are hindered by technology. 15 Mesenchymal stem cells (MSCs) have the advantages of easy expansion in vitro, low immunogenicity, secretion of various active cytokines, and immune regulatory effects, making MSC transplantation for the treatment of acute ischemic stroke a new research focus in recent years.

## Characteristics of Mesenchymal Stem Cells

MSCs have garnered significant attention in the fields of regenerative medicine and tissue engineering due to their unique properties and versatility. Originally identified in the bone marrow, MSCs have since been found in various tissues, including adipose tissue, 16 dental pulp, 17 and umbilical cord. 18 MSCs are multipotent stromal cells capable of differentiating into various cell types. They primarily give rise to cells of the mesodermal lineage, including osteocytes (bone cells), chondrocytes (cartilage cells), and adipocytes (fat cells). MSCs are characterized by their ability to self-renew and generate differentiated progeny, which makes them a critical component of tissue regeneration and repair mechanisms. 19 The original source of MSCs, the bone marrow, provided the foundational understanding of these cells. However, research has revealed that MSCs can be isolated from multiple tissues, including but not limited to: adipose tissue, umbilical cord, dental pulp, etc. 16 17 18 MSCs from adipose tissue was known as adipose-derived stem cells are readily accessible due to the abundance of adipose tissue in the human body. MSCs form umbilical cord contains MSCs that are less likely to carry genetic abnormalities and are often immunologically naïve, making them attractive for transplantation. 20 In addition, dental pulp-derived stem cells have gained attention for their differentiation potential into various cells relevant for dental and craniofacial tissue repair.

### Phenotypic and Molecular Features of Mesenchymal Stem Cells

MSCs exhibit distinct phenotypic characteristics that facilitate their identification and classification. According to the International Society for Cell and Gene Therapy, MSCs must meet several criteria 21 : surface markers, adherent properties, multilineage differentiation potential, and immunomodulatory effects. MSCs express specific surface markers that aid in their identification, including CD73, CD90, and CD105, while lacking hematopoietic markers such as CD34 and CD45. This unique profile allows researchers to differentiate MSCs from other cell types, especially those committed to hematopoietic system. MSCs are adherent to plastic surfaces when cultured in vitro under standard conditions. This characteristic is pivotal for their isolation and expansion, as it allows the cells to proliferate while maintaining their multipotent potential. MSCs can differentiate into various cell types across three primary lineages—mesodermal, ectodermal, and endodermal. This differentiation potential is crucial not only for understanding the molecular mechanisms underlying cell fate decisions but also for developing strategies to apply these cells for therapeutic applications. MSCs possess immunosuppressive properties that enable them to modulate immune responses. 22 They can inhibit the proliferation of T cells 23 24 and the activation of natural killer (NK) cells, making them attractive candidates for applications in treating autoimmune diseases and enhancing graft survival in transplantation settings.

### Functional Characteristics of Mesenchymal Stem Cells

MSCs are a type of multipotent stem cell that are widely present in adult bone marrow, adipose tissue, umbilical cord blood, and other tissues. They have received increasing attention due to their unique biological functions. MSCs not only possess self-renewal ability and multidirectional differentiation potential, but also play important roles in biological processes such as immune regulation and tissue repair. 22 25 26 In clinical applications, MSCs have been explored for the treatment of various diseases, such as acute and chronic inflammation, 27 autoimmune diseases, 28 29 bone and soft tissue injuries, 30 31 due to their low immunogenicity and good safety. In addition, with the rapid development of regenerative medicine, MSCs have a broader application prospect in cardiovascular and cerebrovascular diseases, diabetes, and nervous system diseases.

#### Self-Renewal Capacity and Differentiation Potential

One of the most significant characteristics of MSCs is their self-renewal ability. MSCs can proliferate under specific culture conditions without losing their stem cell characteristics. This characteristic enables them to be extensively amplified in the laboratory, providing abundant cellular resources for subsequent research and clinical applications. Recent studies have shown that signaling pathways such as Wnt, Notch, and Hedgehog play important roles in MSC self-renewal. 32 33 For example, activation of the Wnt signaling pathway can promote MSCs to maintain a self-renewal state, whereas inhibition of this pathway leads to an increased tendency for cell differentiation. The microenvironment of MSCs, including extracellular matrix, adjacent cells, and secreted factors, has a significant impact on their self-renewal ability. 34 By regulating dormancy, metabolic activity, and factors in the microenvironment, MSCs maintain a balance between self-renewal and differentiation. With the development of epigenetics, researchers have found that epigenetic modifications such as DNA methylation and histone modifications also have important effects on the self-renewal ability of MSCs. 35 For example, the methylation status of key genes can directly affect the determination of cell fate, thereby regulating the self-renewal ability of MSCs. In terms of differentiation potential, MSCs can transform into various cell types, including bone cells, chondrocytes, adipocytes, etc. Researches have shown that MSCs can be induced to differentiate into specific types such as cardiomyocytes and neurons under appropriate conditions. 36 37 This multidirectional differentiation mechanism has opened up new directions for regenerative medicine, especially in tissue engineering and regenerative therapy.

#### Immune Regulatory Function

Another important biological function of MSCs is their immune regulatory ability. MSCs can regulate immune responses by secreting various bioactive factors, such as cytokines, chemokines, and extracellular vesicles. MSCs can inhibit the activity of various immune cells, including T cells, B cells, NK cells, and macrophages, thereby providing protection against autoimmune diseases and transplant rejection. 23 38 The immunomodulatory effect of MSCs is believed to be closely related to their application in specific disease states. For example, in the treatment of autoimmune diseases such as systemic lupus erythematosus 39 40 and rheumatoid arthritis, 41 MSCs have shown good clinical efficacy due to their regulation of the immune system. This feature provides new ideas for the application of MSCs in the fields of cell therapy and gene therapy.

#### Promote Tissue Repair and Regeneration

MSCs play an important role in promoting tissue repair and regeneration processes. MSCs can not only differentiate into cells with various specialized functions, but also promote the regeneration of damaged tissues by releasing growth factors (such as hepatocyte growth factor [HGF], vascular endothelial growth factor [VEGF], and fibroblast growth factor) and ECM components. 42 These factors can stimulate the proliferation, migration, and differentiation of endogenous cells, thereby accelerating wound healing and tissue repair. The application of MSCs has also received attention in the treatment of cardiovascular and joint diseases. Research has shown that injecting MSCs into damaged myocardial tissue can improve heart function. 43 In bone repair such as fractures and bone defects, the application of MSCs helps with bone generation and regeneration. These research findings provide new treatment strategies for clinical practice and promote the development of regenerative medicine.

## The Mechanism of Mesenchymal Stem Cells in Treating Ischemic Stroke

The mechanism MSCs in the treatment of ischemic stroke has gradually attracted widespread attention, mainly reflected in their multidirectional differentiation ability, immune regulatory effects, and neuroprotective effects. MSCs can differentiate into neurons, glial cells, and endothelial cells, thereby promoting the repair and regeneration of damaged brain tissue. 37 In addition, MSCs exert significant anti-inflammatory effects by secreting various cytokines and bioactive molecules, such as pre-glial cell-derived neurotrophic factor, transforming growth factor-β (TGF-β), etc., inhibiting neuronal apoptosis and reducing inflammation caused by ischemia. Meanwhile, MSCs also help to enhance angiogenesis, improve local blood circulation, and further promote reperfusion and recovery in ischemic areas of the brain. 44 Through the above comprehensive mechanisms, MSCs have shown promising prospects in the treatment of ischemic stroke and have become an important research direction in the field of regenerative medicine.

### Immune Regulation

MSCs can inhibit inflammatory response, improve local microenvironment, and alleviate secondary damage caused by ischemia. Ischemic brain injury is mainly caused by insufficient blood flow to the brain, resulting in hypoxia of brain tissue and lack of nutrients. This process can trigger a series of pathological changes, including cellular metabolic disorders, increased intracellular calcium ion concentration, and increased production of reactive oxygen species (ROS). 45 46 These changes further lead to cell apoptosis and necrosis. More seriously, ischemic brain tissue can initiate an inflammatory response, releasing a large amount of inflammatory mediators such as cytokines and chemokines, leading to further damage to surrounding normal brain tissue, a phenomenon known as secondary injury. The key role of inflammatory response in ischemic brain injury has been widely approved. 47 48 After ischemia, the infiltration of inflammatory cells, especially the aggregation of monocytes and neutrophils, can lead to the release of inflammatory mediators, thereby exacerbating the damage to the local microenvironment. In addition, the activation of microglia and astrocytes in the brain can further promote inflammatory responses, forming a vicious cycle. 49 Therefore, inhibiting inflammatory response and improving the microenvironment are important goals for treating ischemic brain injury. MSCs are a type of adult stem cell with self-renewal ability and multipotent differentiation potential, widely present in sources such as bone marrow, adipose tissue, and umbilical cord. Compared with other types of stem cells, MSCs have good immune regulatory ability. MSCs can exert protective effects after tissue damage by releasing various cytokines, anti-inflammatory substances, and growth factors.

The immune regulatory mechanisms of these cells mainly include: (1) secreting anti-inflammatory cytokines such as interleukin-10 (IL-10) and TGF-β to inhibit the function of inflammatory cells 50 51 ; (2) directly regulate the activity of immune cells by expressing programmed cell death ligand 1 (PD-L1) and cytokines 52 ; (3) promote the repair and regeneration of local endogenous cells. Therefore, the application of MSCs after ischemic brain injury has shown great potential. Preliminary studies have shown that MSCs can effectively inhibit the inflammatory response after ischemic brain injury. 53 54 The injection of MSCs into ischemic lesions can significantly reduce the infiltration of inflammatory cells and inhibit the excessive release of inflammatory mediators. The specific mechanism includes enhancing M2 polarization of macrophages and inhibiting M1 activation. In addition, MSCs can reduce oxidative stress in ischemic brain tissue and alleviate inflammatory reactions by releasing various factors such as HGF and prostaglandin E2. 55

Research has shown that MSCs can significantly reduce the expression of proinflammatory factors such as tumor necrosis factor-α and IL-6 in ischemic brain tissue, while increasing the level of anti-inflammatory factor IL-10. 51 The implementation of this anti-inflammatory effect can not only reduce brain tissue damage, but also promote the survival and functional recovery of neurons. MSCs can improve the microenvironment of ischemic brain tissue through various pathways. First, MSCs can secrete neurotrophic factors such as brain-derived neurotrophic factor (BDNF) and neurotrophin-3, which can effectively support the survival and growth of nerve cells, promote neural development and regeneration. 56 Second, MSCs also have significant antioxidant effects, which can reduce the production of ROS and mitigate cellular damage caused by oxidative stress after ischemia. In addition, MSCs improve the microenvironment and promote tissue repair by promoting angiogenesis and enhancing local blood flow, increasing the supply of oxygen and nutrients to nerve cells.

### Promoting Angiogenesis

MSCs promote neovascularization and improve local blood supply by secreting signaling molecules such as vascular growth factors. Research has shown that the role of MSCs in ischemic brain tissue is not only limited to cell replacement, but also promotes the formation of new blood vessels by secreting signaling molecules such as VEGF, thereby improving local blood supply and promoting the recovery of damaged brain tissue. 57 After an ischemic attack, brain tissue is deprived of oxygen and nutrients due to insufficient blood flow. In this case, brain cells gradually enter a state of metabolic imbalance and eventually die irreversibly. Ischemic injury is usually accompanied by a series of inflammatory reactions, further exacerbating the damage to brain tissue. Research has shown that the regenerative ability after ischemia is limited by the environment after injury, and the lack of nourishment from new blood vessels is one of the key factors limiting therapeutic efficacy. 58 MSCs are a type of adult stem cell with self-renewal and multipotent differentiation potential, widely present in various tissues. They can not only differentiate into adipocytes, chondrocytes, and fibroblasts, but also have excellent immune regulatory and hydroxyl radical scavenging abilities. Recent studies have shown that MSCs can also promote tissue repair and regeneration by secreting various growth factors and cytokines. 59 Among them, VEGF is one of the important vascular growth factors secreted by MSCs. VEGF plays a crucial role in the process of angiogenesis, as it can promote endothelial cell proliferation and migration, induce basement membrane degradation, and thus facilitate the formation and maturation of vascular lumens. In addition, MSCs can secrete various other factors that promote angiogenesis, such as matrix metalloproteinases (MMPs), epidermal growth factor, etc., which further enhance the efficiency of angiogenesis through synergistic effects. 60

In recent years, more and more researches have focused on the application of MSCs in ischemic brain injury. 61 62 Research has found that MSC transplantation can significantly improve blood supply and functional recovery after ischemic injury. 63 By injecting MSCs, the vascular density in the damaged area can be effectively increased, the infiltration of inflammatory cells can be reduced, cell apoptosis can be inhibited, and nerve regeneration can be promoted. Especially in the early stage after ischemia, the injection of MSCs can significantly increase the expression level of VEGF in the ischemic area, thereby promoting angiogenesis, improving blood supply, and providing necessary environment and conditions for the repair of ischemic brain tissue. 64 This mechanism of improving brain tissue recovery by enhancing local blood supply provides a new approach for the clinical treatment of ischemic brain injury.

### Antioxidant Effect

Research has shown that MSCs can reduce the concentration of ROS after ischemic brain tissue injury, thereby alleviating oxidative stress damage to nerve cells. 59 In normal physiological processes, ROS are byproducts of cellular metabolism, including superoxide, hydroperoxide, and hydroxyl radicals. Moderate levels of ROS participate in cell signaling and immune responses, but their concentration often increases significantly in ischemic brain injury, leading to significant oxidative stress. Oxidative stress refers to the imbalance between oxidants and antioxidants in the body, where ROS attack cell membranes, proteins, and DNA, ultimately leading to cell death. In ischemic brain injury, oxidative stress causes significant damage to nerve cells via activation of PI3K/AKT/Nrf2/HO-1 pathway, leading to cell apoptosis and tissue dysfunction. 65 Research has found that MSCs not only promote tissue regeneration by secreting bioactive factors and extracellular vesicles, but also alleviate cell damage through antioxidant effects. 66 One of the mechanisms of action of MSCs after ischemic brain tissue injury is to alleviate oxidative stress by reducing the concentration of ROS. MSCs can secrete various antioxidant factors, such as superoxide dismutase, glutathione peroxidase, and antioxidant enzymes, which can effectively eliminate ROS in the body. In addition, MSCs can protect nerve cells by enhancing the antioxidant capacity of host cells and promoting the expression of antioxidant enzymes. 67

### Promoting Cell Survival

Ischemic stroke can induce autophagy, apoptosis, and necrosis of cells. Studies have shown that MSCs can regulate cell death after ischemic stroke, promote cell survival, and reduce neurological damage. 68 First, transplantation of MSCs can increase autophagy in the early stage (within 24 hours after ischemia) and inhibit autophagy in the late stage (48–72 hours after ischemia), promoting the survival of damaged cells. In the early stage after stroke, MSCs secrete BDNF, reduce the activation of the mTOR pathway, promote the expression of autophagy markers Beclin-1 and microtubule associated protein LC3, enhance neuronal autophagy, inhibit neuronal apoptosis, and ultimately improve the recovery of cerebral ischemic injury. 69 However, prolonged hypoxia and glucose deficiency lasting 48 to 72 hours or longer can lead to excessive autophagy. During this period, MSCs transplantation can reduce the expression of Beclin-1 and LC3, promote neurite outgrowth and regeneration, inhibit autophagy, and protect neurons. In the MCAO rat model, tail vein injection of adipose-derived MSCs can induce the activation of anti-apoptotic factor Bcl-2, inhibit endoplasmic reticulum stress, and the activity of pro-apoptotic molecule Bax, thereby reducing apoptosis in ischemic brain tissue. 70 MSCs can also cause dysfunction of MMPs, prevent their upregulation, inhibit cell death signals such as caspase-3, and activate cell survival signaling pathways such as STAT3 and Akt phosphorylation. Recently, it has been found that MSCs can rescue damaged neurons by transferring their own mitochondria to neurons, increasing neuronal activity, and improving metabolic function. 71

## Conclusion

MSCs therapy for ischemic stroke has shown promising application prospects, but still faces multiple challenges such as efficacy evaluation, heterogeneity of the MSCs source and molecular mechanism. Establishing a systematic framework to develop a more comprehensive and in-depth understanding of the therapeutic potential and application limitations of MSCs, can provide more effective strategies for the treatment of ischemic stroke. With the continuous deepening of research, MSCs are expected to become important means of treating ischemic stroke.
